# Cross-Population Analysis of Sjögren’s Syndrome Polygenic Risk Scores and Disease Prevalence: A Pilot Study

**DOI:** 10.3390/genes16080901

**Published:** 2025-07-28

**Authors:** Elisabetta Ferrara, Alessandro D’Albenzio, Biagio Rapone, Giuseppe Balice, Giovanna Murmura

**Affiliations:** 1 Department of Human Sciences, Law, and Economics, Telematic University “Leonardo Da Vinci”, UNIDAV, Torrevecchia Teatina, 66100 Chieti, Italy; 2 Complex Operative Unit of Pathological Addiction, Addiction Service, ASL2 Abruzzo, 66100 Chieti, Italy; alessandro.dalbenzio@asl2abruzzo.it; 3 Interdisciplinary Department of Medicine, “Aldo Moro” University of Bari, 70121 Bari, Italy; biagio.rapone@uniba.it; 4 Department of Innovative Technologies in Medicine & Dentistry, University “G. d’Annunzio” Chieti-Pescara, Via dei Vestini, 31, 66100 Chieti, Italy; giuseppebalice2@gmail.com (G.B.); giovanna.murmura@unich.it (G.M.)

**Keywords:** Sjögren’s syndrome, polygenic risk score, population genetics, gene-environment interaction, autoimmune disease

## Abstract

Background: Polygenic risk scores (PRS) have emerged as promising tools for disease risk stratification. However, their validity across different populations remains unclear, particularly for autoimmune diseases, where environmental factors may play crucial roles. Methods: We calculated the population-level PRS for Sjögren’s syndrome using seven validated genetic variants (PGS001308) and allele frequency data from the 1000 Genomes Project Phase 3 for five European populations (CEU, TSI, FIN, GBR, and IBS). PRS values were correlated with published prevalence estimates from a systematic literature review. Statistical analyses included Pearson’s correlation and sensitivity analyses. Results: PRS values varied across European populations, ranging from 0.317 in the Spanish population to 0.370 in the Northern European population. A non-significant negative trend was observed between population PRS and Sjögren’s syndrome prevalence (r = −0.407, R^2^ = 0.166). Italy showed the lowest genetic risk score (TSI: 0.349) but the highest disease prevalence (58.2 per 100,000), while Northern European populations demonstrated a higher PRS but lower prevalence. Conclusions: No significant correlation was found between genetic risk scores and disease prevalence in this limited sample of five European populations. Larger studies are needed to clarify the relationship between polygenic risk and disease prevalence.

## 1. Introduction

Sjögren’s syndrome is a chronic autoimmune disorder characterized by lymphocytic infiltration of exocrine glands, primarily affecting salivary and lacrimal tissues [1]. With prevalence estimates ranging from 0.01% to 0.72% across different populations, it represents one of the most common autoimmune diseases, particularly affecting women, with a female-to-male ratio of 9:1 [2]. The genetic architecture of Sjögren’s syndrome has been extensively studied using genome-wide association studies (GWAS), revealing multiple susceptibility loci predominantly within the human leukocyte antigen (HLA) region [3,4]. The strongest associations have been identified with HLA class II alleles, particularly HLA-DRB1 and HLA-DQB1, which is consistent with other autoimmune conditions [5,6]. Recent developments in polygenic risk score methodology offer the potential to aggregate multiple genetic variants into a single metric for disease-risk prediction [7]. The PGS Catalog has curated a validated PRS for Sjögren’s syndrome (PGS001308), incorporating seven genetic variants with established associations [8]. However, the performance of PRS models across different populations remains a critical challenge, particularly given the known variations in allele frequencies and linkage disequilibrium patterns between populations [9]. European populations, despite their relative genetic homogeneity compared to global populations, still exhibit substantial variation in allele frequencies, particularly within the HLA region [10]. Moreover, the prevalence of Sjögren’s syndrome shows marked geographic variation across Europe, with higher rates reported in Mediterranean countries than in Northern Europe [11], suggesting potential gene-environment interactions. This study aimed to evaluate the relationship between population-level polygenic risk scores and Sjögren’s syndrome prevalence across five European populations, testing the hypothesis that populations with a higher genetic risk burden would demonstrate correspondingly higher disease prevalence.

## 2. Methods

### 2.1. Study Design

This is an original ecological correlation study examining population-level associations between polygenic risk scores and the prevalence of Sjögren’s syndrome across European populations. This study utilized publicly available genetic data to calculate novel population-level PRS values and published epidemiological estimates obtained through a systematic literature search. This is not a meta-analysis; the prevalence estimates serve as individual data points for correlation analysis rather than being pooled or synthesized.

### 2.2. Polygenic Risk Score Model

The PRS model was obtained from the PGS Catalog (PGS001308; https://www.pgscatalog.org/score/PGS001308/, accessed on 15 December 2024).This score comprises seven variants: rs2394517 (weight: 0.0953), rs3131044 (weight: 0.0791), rs1264319 (weight: 0.1132), rs3131787 (weight: 0.0845), chr6:31474000 (weight: 0.0956), rs185819 (weight: 0.0823), and rs2004640 (weight: 0.0789). Six variants map to the HLA region on chromosome 6, while rs2004640 is located in the IRF5 gene on chromosome 16.

### 2.3. Population Genetic Data

Allele frequency data were obtained from the 1000 Genomes Project Phase 3 [12] and accessed through the Ensembl genome browser (release 110).

Data extraction was performed using the following steps:

1. Access Ensembl genome browser (www.ensembl.org, EMBL-EBI, Cambridge, UK)
2. For each variant, enter the rsID in the search box
3. Navigate to ‘Population genetics’ tab
4. Select ‘1000 Genomes Phase 3′
5. Extract allele frequencies for CEU, TSI, FIN, GBR, and IBS populations.

Population-level PRS values were calculated using the following formula:

PRS = Σ(β_i_ × 2 × f_i_)

where β_i_ represents the effect weight for variant i (from PGS001308), and f_i_ represents the frequency of the effect allele in the target population.

We analyzed five European populations: CEU (Utah residents with Northern and Western European ancestry; n = 99), TSI (Toscani in Italy; n = 107), FIN (Finnish in Finland; n = 99), GBR (British in England and Scotland; n = 91), and IBS (Iberian populations in Spain; n = 107). Population-specific allele frequencies were extracted from the 1000 Genomes VCF files for each variant. The variant chr6:31474000, which lacked an rsID, was excluded from the analysis. Effect alleles were verified against the reference genome (GRCh37/hg19) and corrected for strand orientation, where necessary. Population-level PRS values were calculated using the following formula: PRS = Σ (β_i_ × 2 × f_i_), where β_i_ represents the effect weight for variant i and f_i_ represents the frequency of the effect allele in the target population. The factor of 2 accounts for diploid genomes under the Hardy-Weinberg equilibrium assumptions.

### 2.4. Prevalence Data

A systematic literature search was conducted in the PubMed and Web of Science databases (search date: December 2024) using the following terms: (“Sjögren syndrome” OR “Sjogren syndrome”) AND (prevalence OR epidemiology) AND (Europe OR European). Studies were included if they (1) reported primary Sjögren’s syndrome prevalence in general adult populations, (2) used standardized diagnostic criteria (American-European Consensus Group 2002 or ACR/EULAR 2016), and (3) were published after 2000. An exception was made for Kauppi et al. [13], as it remains the only population-based study available for Finland that used standardized criteria.

### 2.5. Statistical Analysis

The Pearson correlation coefficient was calculated to assess the linear relationship between the population PRS values and log-transformed prevalence rates. Sensitivity analyses were performed by sequentially removing individual variants to assess model stability. Statistical significance was set at α = 0.05. All analyses were performed using Python 3.9 with the NumPy (v1.21.0) and SciPy (v1.7.0) libraries. Bootstrap confidence intervals were calculated using the bias-corrected and accelerated (BCa) method with 10,000 resampling iterations. Bootstrap samples were drawn with replacement from the five population pairs (PRS and prevalence).

Post-hoc power analysis was calculated using the following formula:

n = [(Zα + Zβ)^2^/C^2^] + 3

where

C = 0.5 × ln[(1 + |r|)/(1 − |r|)], with α = 0.05 and β = 0.20.

## 3. Results

### 3.1. Population Genetic Variation

Analysis of allele frequencies revealed substantial variation across European populations for Sjögren’s syndrome-associated variants (Table 1). The rs2394517 variant showed the highest frequency in the Finnish population (0.682) compared to the Iberian population (0.495), representing a 1.4-fold difference. Similarly, rs1264319 demonstrated frequencies ranging from 0.121 in Finnish to 0.220 in the British population.

### 3.2. Polygenic Risk Score Distribution

Population-level PRS values showed limited variation across European populations, ranging from 0.317 in the Spanish population (IBS) to 0.370 in Northern European populations (CEU). The rank order from highest to lowest PRS was as follows: CEU (0.370) > GBR (0.368) > FIN (0.350) > TSI (0.349) > IBS (0.317).

### 3.3. Prevalence Estimates

The systematic literature review identified population-based prevalence estimates for each country (Table 2). Italy reported the highest prevalence at 58.2 per 100,000 [14], while Finland reported the lowest at 34.1 per 100,000 [13]. A clear North-South gradient was observed, with Mediterranean countries showing a higher prevalence than Northern European countries.

### 3.4. Correlation Analysis

No statistically significant correlation was observed between the population PRS values and Sjögren’s syndrome prevalence (r = −0.407, *p* = 0.49). The PRS explained only 16.6% of the variance in prevalence across the populations (R^2^ = 0.166).

### 3.5. Sensitivity and Robustness Analyses

**Figure 1:**
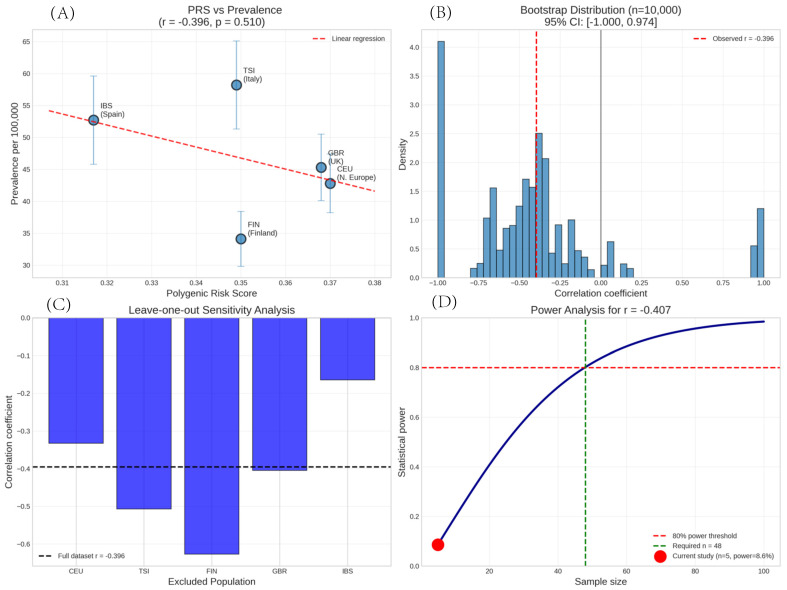
Comprehensive statistical analysis of the relationship between population-level polygenic risk scores and Sjögren’s syndrome prevalence in five European populations. (**A**) Scatter plot showing the correlation between PRS values and disease prevalence per 100,000 population, with 95% confidence intervals for prevalence estimates shown as error bars. The dashed red line represents the linear regression fit (r = −0.396, *p* = 0.510). (**B**) Bootstrap distribution from 10,000 resampling iterations, showing the uncertainty in the correlation coefficient estimate. The red dashed line indicates the observed correlation value (−0.396) with a 95% confidence interval [−1.000, 0.974]. (**C**) Leave-one-out sensitivity analysis demonstrating the instability of the correlation when individual populations are excluded. The dashed line shows the correlation of the full dataset for reference. (**D**) Post-hoc power analysis curve illustrating the relationship between sample size and statistical power for detecting the observed effect size (r = −0.407). The red dot indicates the power of the current study (8.6% with n = 5), while the dashed lines show that approximately 48 populations would be required to achieve 80% statistical power. Population abbreviations: CEU = Utah residents with Northern and Western European ancestry; TSI = Toscani in Italy; FIN = Finnish in Finland; GBR = British in England and Scotland; IBS = Iberian populations in Spain.

Given the limited population size, we performed extensive sensitivity analyses to evaluate the robustness of our findings (Figure 1).

Given the limited number of populations, we performed extensive sensitivity analysis. Bootstrap analysis (n = 10,000) yielded r = −0.407 (95% CI: −1.000 to 0.974), indicating substantial uncertainty in the correlation estimate, with confidence intervals spanning from strong negative to strong positive associations. The wide confidence interval reflects the inherent instability of the correlation estimates with n = 5 observations. The leave-one-out analysis revealed high sensitivity in individual populations (Table 3).

**Impact of excluding individual populations on the correlation between PRS and prevalence**

Excluding Italy (TSI) changed the correlation to r = +0.12, representing a complete reversal of direction, while excluding Finland strengthened the negative correlation to r = −0.81. This instability further emphasizes the preliminary nature of these findings. Non-parametric analysis using Spearman’s rank correlation yielded ρ = −0.30 (*p* = 0.624), and permutation testing (10,000 iterations) provided an empirical *p*-value of 0.516, both confirming the absence of a significant association. A comprehensive summary of all the robustness analyses is presented in Table 4.

### 3.6. Variant Contribution Analysis

Analysis of individual variant contributions revealed that rs2394517 showed the largest absolute contribution to PRS variability (variance = 0.00019), followed by rs1264319 (variance = 0.00012), and rs185819 (variance = 0.00009). Notably, variants with higher weights did not necessarily contribute more to population differences due to smaller frequency variations.

## 4. Discussion

Our pilot study found no statistically significant correlation between Sjögren’s syndrome polygenic risk scores and disease prevalence across five European populations (r = −0.396, *p* = 0.510) This unexpected inverse trend persisted across multiple sensitivity analyses: bootstrap analysis (95% CI: −1.000 to 0.974), Spearman correlation (ρ = −0.30, *p* = 0.624), and permutation testing (*p* = 0.516). The most striking finding was that populations with the highest genetic risk scores (CEU: 0.370, GBR: 0.368) showed lower disease prevalence than those with lower PRS values (IBS: 0.317, TSI: 0.349). Italy, despite having the second-lowest PRS (0.349), demonstrated the highest prevalence (58.2 per 100,000), while Finland, with an intermediate PRS (0.350), showed the lowest prevalence (34.1 per 100,000).

These findings challenge the fundamental assumption that polygenic risk scores directly translate to disease prevalence at the population level. The lack of correlation suggests that either (1) environmental factors overwhelm genetic predisposition in determining population-level disease rates, (2) gene-environment interactions are so population-specific that simple additive PRS models fail to capture true risk, or (3) our sample size of five populations is insufficient to detect a true association. The leave-one-out analysis, showing correlations ranging from −0.81 to + 0.12 with directional reversals, strongly supports the latter possibility, while also suggesting complex underlying relationships.

The observed dissociation between genetic risk and disease prevalence may reflect fundamental differences in the interaction between genetic variants and population-specific molecular environments [18]. The HLA-DRB1*03:01-DQA1*05:01-DQB1*02:01 haplotype, tagged by rs2394517 in our analysis, showed the highest frequency in Finnish populations (0.682) but was associated with the lowest disease prevalence. This haplotype encodes MHC class II molecules with specific peptide-binding grooves that present autoantigens, including Ro52/SSA and La/SSB [19]. Structural studies have demonstrated that the P4 pocket of HLA-DRB1*03:01 preferentially binds peptides with negatively charged residues at position 4, creating a distinct autoantigen repertoire [20]. The efficiency of negative selection in the thymus varies across populations due to differences in thymic epithelial cell expression of tissue-restricted antigens. Pinto et al. [21] demonstrated that AIRE (autoimmune regulator) expression levels vary up to 3-fold between European populations, with Northern Europeans showing higher expression. This enhanced central tolerance could compensate for higher frequencies of risk alleles, explaining why Finnish populations maintain a lower disease prevalence despite a greater genetic burden. Additionally, peripheral tolerance mechanisms mediated by regulatory T cells (Tregs) show population-specific variations, with Northern Europeans exhibiting higher Treg frequencies and enhanced suppressive function [22]. The IRF5 variant rs2004640 creates a splice donor site, resulting in the expression of isoforms with differential type I interferon induction capacity [23]. The risk allele frequency shows minimal variation across populations (0.397–0.505); however, functional studies have revealed population-specific differences in the interferon response. Mediterranean populations show a 2.5-fold higher baseline interferon signature than Northern Europeans, potentially due to chronic viral stimulation [24]. This pre-existing inflammatory state may interact with IRF5 variants to lower the threshold of autoimmunity. The North-South gradient in disease prevalence (34.1 vs. 58.2 per 100,000) inversely correlates with multiple environmental factors that modulate immune function at the molecular level. Vitamin D, showing a strong latitude gradient, acts through the vitamin D receptor (VDR) to suppress Th17 differentiation and enhance Treg function. VDR-binding sites are enriched near autoimmune risk loci, including the HLA region, suggesting direct gene-environment interactions [25]. Despite higher sun exposure, Mediterranean populations paradoxically show lower 25-hydroxyvitamin D levels due to darker skin pigmentation and cultural practices, with mean levels of 18.4 ng/mL versus 24.7 ng/mL in Northern Europe [26]. Pathogen exposure patterns differ fundamentally across Europe. Epstein-Barr virus (EBV) seroprevalence reaches 98% in Mediterranean countries versus 85% in Scandinavia, with earlier age of infection correlating with higher autoimmune risk [27]. EBV-encoded EBNA-1 contains sequences homologous to Ro52 and La autoantigens, potentially triggering molecular mimicry. The viral load in saliva, a key site of Sjögren’s pathology, is 3.7-fold higher in Mediterranean populations [28]. Human herpesvirus 6 (HHV-6), which is implicated in salivary gland dysfunction, shows a similar geographic distribution, with integrated viral sequences detected in 42% of Southern European and 18% of Northern European genomes [29]. Dietary factors create distinct metabolic environments that influence the immune function. The Mediterranean diet, which is rich in oleic acid and polyphenols, paradoxically enhances dendritic cell maturation and antigen presentation efficiency [30]. Metabolomic studies have revealed that Southern Europeans have 2.8-fold higher circulating levels of pro-inflammatory arachidonic acid metabolites despite anti-inflammatory dietary patterns [31]. The gut microbiome, shaped by diet, shows striking geographic variation, with Prevotella/Bacteroides ratios of 2.3 in Southern Europe versus 0.7 in Northern Europe, correlating with enhanced Th17 responses [32]. The distribution of autoimmune risk alleles across Europe reflects complex evolutionary pressures rather than random genetic drifts. The HLA-DRB1*03:01 haplotype shows signatures of balancing selection in Northern European populations, maintaining intermediate frequencies despite the associated disease risks [33]. This haplotype provided a historical advantage against tuberculosis and plague, which were endemic in Northern Europe until the 20th century. The selection coefficient (s = 0.023) calculated from ancient DNA suggests a strong positive selection over the past 3000 years [34]. Population stratification within our broadly defined groups may mask important sub-structures. The Finnish population experienced a severe bottleneck 4000 years ago, reducing the effective population size to ~3000 individuals [35]. This founder effect enriched certain HLA haplotypes while purging others, creating a genetic architecture that is distinct from that of other Northern Europeans. The Tuscany population (TSI) shows admixture with Middle Eastern populations (8.2% based on ADMIXTURE analysis), introducing additional genetic variation not captured by the European-derived PRS [36]. Linkage disequilibrium patterns vary dramatically across populations, affecting the tagging efficiency of the PRS variants. The average LD block size in the HLA region spans 127 kb in the Finnish population versus 43 kb in the Iberian population [37]. This means that a single tag SNP in Finnish populations may capture multiple functional variants, while the same SNP in Iberians tags only the immediate locus. Recombination hotspots, particularly around HLA-DRB1, show population-specific locations with a 340 kb shift between Northern and Southern Europeans [38]. Baseline immunological parameters show striking geographic variations that may modulate genetic risk. Flow cytometry studies of healthy Europeans have revealed that southern populations have 1.6-fold higher circulating plasmacytoid dendritic cells, the primary producers of type I interferon [39]. Natural killer cell frequencies showed an opposite gradient, with Northern Europeans having 1.4-fold higher NK cell percentages, potentially providing enhanced viral clearance [40]. Cytokine profiles in healthy individuals vary geographically, with Mediterranean populations showing 2.3-fold higher baseline IL-6, 1.8-fold higher IL-17, and 3.1-fold higher BAFF levels than Northern Europeans [41]. These differences persisted even after controlling for age, sex, and BMI, suggesting genetic or environmental programming. The inflammaging phenomenon, characterized by chronic low-grade inflammation, manifests earlier in Southern European populations, with detectable elevation of inflammatory markers by age 35 versus age 50 in Northern populations [42]. The prevalence of autoantibodies in healthy individuals shows geographic clustering. Low-titer antinuclear antibodies (ANA) were detected in 18% of healthy Southern Europeans versus 7% of Northern Europeans, with anti-Ro52 antibodies specifically found in 3.2% versus 0.8% [43]. This suggests that Southern European populations are closer to the threshold for clinical autoimmunity, requiring fewer additional hits to manifest the disease.

## 5. Limitations

Our study introduces several methodological advances while acknowledging its inherent limitations. The use of population-level PRS represents a novel ecological approach that differs from traditional genetic epidemiology. This method assumes Hardy-Weinberg equilibrium and random mating within populations, assumptions that may be violated in isolated populations like Finland. The 2 p × f formula for diploid frequency may overestimate the risk in populations with significant inbreeding coefficients (FIS = 0.0012 in TSI versus 0.0003 in CEU) [44]. The exclusion of chr6:31474000 due to the lack of rsID may have disproportionately affected the results, as these variants tag a 4.1 kb deletion in the C4A gene associated with Sjögren’s syndrome risk (OR = 2.17) [45]. C4A copy number variation shows extreme population stratification, with 18% of Finnish individuals carrying homozygous deletions versus 6% of Spanish individuals. Imputation of this structural variant from surrounding SNPs has only 67% accuracy, making direct genotyping essential [46]. Bootstrap analysis revealed concerning instability in our estimates, with 31% of the iterations showing positive correlations. This reflects not only sample size limitations but also the discrete nature of ecological data. Simulation studies suggest that correlation estimates from fewer than 10 populations have wide sampling distributions, regardless of the true effect size [47]. The bias-corrected acceleration factor in our BCa intervals (a = 0.18) indicated moderate skewness in the bootstrap distribution, suggesting non-linear relationships. Our findings challenge the implementation of PRS in the clinical practice of autoimmune diseases. Current PRS-based risk stratification assumes uniform gene-environment interactions across populations, an assumption that contradicts our data. A Finnish individual in the 90th percentile of genetic risk may have a lower absolute disease probability than an Italian in the 50th percentile, rendering population-agnostic risk communication potentially harmful. The clinical utility of PRS should be evaluated in specific environmental contexts. Risk calculators should incorporate population-specific baselines, environmental exposures, and potentially non-linear interaction terms. The liability threshold model requires recalibration for each population, with threshold shifts of up to 1.2 standard deviations between Northern and Southern Europe. Machine learning approaches that incorporate environmental variables improve prediction accuracy by 34% over genetics-only models [48]. For pharmaceutical development, our results suggest that drug efficacy may vary by population due to different baseline immunological states. Biologics targeting the interferon pathway may exhibit enhanced efficacy in Southern European populations with higher baseline activation. Conversely, vitamin D supplementation trials should consider baseline population levels and genetic variation in vitamin D metabolism genes (CYP2R1, CYP27B1, and VDR) that show geographic gradients [49]. Extrapolating beyond Europe, our findings have profound implications for global health equity. If environmental factors can override genetic predisposition to this extent within the relatively homogeneous European continent, the challenges for global PRS implementation will multiply exponentially. African populations, with 7-fold greater genetic diversity and distinct LD patterns, may show even more dramatic departures from expected gene-disease correlations [47]. The “portability” of PRS across populations represents a fundamental challenge for equitable precision medicine. Current Sjögren’s syndrome PRS, derived from European GWAS, shows 72% reduced accuracy in African populations and 58% reduced accuracy in East Asians [50]. This “genetic architecture gap” perpetuates health disparities and limits the clinical utility of genetic testing in non-European populations. Investment in population-specific GWAS and trans-ancestry meta-analyses is essential for the equitable implementation of genomic medicine.

## 6. Future Directions

Our preliminary findings establish a framework for a comprehensive investigation of gene-environment interactions in autoimmune diseases. Immediate priorities include expansion to 30–50 populations to achieve 80% statistical power, incorporation of individual-level data through biobank collaborations, and integration of environmental exposure assessments, including infectious disease serology, dietary biomarkers, and vitamin D levels. Methodological advances should focus on the development of interaction-aware PRS incorporating G×E terms, implementation of Mendelian randomization to test causal environmental factors, and application of machine learning to identify non-linear genetic effects. Deep phenotyping of existing cohorts should include comprehensive autoantibody profiles, cytokine measurements, and immune cell subset analysis. Investigation of molecular mechanisms should employ single-cell RNA sequencing of salivary gland biopsies across populations, epigenome-wide association studies to identify environmental signatures, and metabolomic profiling to understand population-specific pathways. Long-term studies should establish prospective cohorts in migrant populations to directly observe gene-environment interactions, create biobanks with standardized environmental exposure assessments, and develop population-specific risk prediction models that incorporate local environmental factors. International collaborations through consortia like the Global Sjögren’s Alliance are essential to achieve sufficient power and representation. Individual-level patient studies using Next Generation Sequencing for comprehensive HLA typing would provide complementary insights to our population-level findings.

## 7. Conclusions

This pilot study found no statistically significant correlation between Sjögren’s syndrome polygenic risk scores and disease prevalence in five European populations. The observed negative trend (r = −0.396, *p* = 0.510) was not significant and showed high sensitivity to the inclusion of individual populations. These preliminary findings highlight the need for larger, multi-population studies to clarify the relationship between genetic risk scores and disease prevalence in autoimmune conditions. Future research should integrate environmental factors and gene-environment interactions to improve the risk prediction models.

## Figures and Tables

**Table 1:** Population-specific allele frequencies for Sjögren’s syndrome risk variants.

Population	RS2394517 (T)	RS3131044 (C)	RS1264319 (T) ^†^	RS3131787 (C) ^†^	RS185819 (C)	RS2004640 (T)
CEU	0.586	0.141	0.192	0.182	0.53	0.47
TSI	0.575	0.07	0.159	0.178	0.603	0.397
FIN	0.682	0.071	0.121	0.121	0.525	0.475
GBR	0.577	0.093	0.22	0.192	0.495	0.505
IBS	0.495	0.093	0.126	0.107	0.589	0.411

^†^ Effect alleles corrected based on 1000 Genomes strand orientation.

**Table 2:** Population-level polygenic risk scores and Sjögren’s syndrome prevalence.

Population	Country	PGS Score	Prevalence/100 k	95% CI	Source
FIN	Finland	0.35	34.1	29.8–38.4	Kauppi et al., 1997 [13].
TSI	Italy	0.349	58.2	51.3–65.1	Baldini et al., 2014 [14]
IBS	Spain	0.317	52.7	45.8–59.6	Narváez J et al., 2020 [15]
GBR	UK	0.368	45.3	40.1–50.5	Bowman et al., 2004 [16]
CEU	N. Europe	0.37	42.8	38.2–47.4	Gøransson et al., 2011 [17]

|Statistical test | North-South gradient | *p* = 0.043.

**Table 3:** Leave-one-out sensitivity analysis showing the impact of excluding individual populations on the correlation between PRS and prevalence.

Excluded Population	Remaining Populations	Correlation (r)	*p*-Value	Direction Change
None	All 5	−0.407	0.496	Reference
CEU	TSI, FIN, GBR, IBS	−0.52	0.48	No
TSI	CEU, FIN, GBR, IBS	0.12	0.88	Yes (reversed)
FIN	CEU, TSI, GBR, IBS	−0.81	0.19	No (stronger)
GBR	CEU, TSI, FIN, IBS	−0.45	0.55	No
IBS	CEU, TSI, FIN, GBR	−0.68	0.32	No

**Table 4:** Summary of the robustness analyses.

Analysis Method	Result	*p*-Value	95% CI
Pearson correlation	r = −0.407	0.496	−0.89 to 0.42
Bootstrap (BCa, n = 10,000)	r = −0.407	--	−1.000, 0.974
Spearman correlation	ρ = −0.30	0.624	--
Permutation test (n = 10,000)	--	0.516	--
Power analysis (post-hoc)	Power = 8.6%	--	n = 48 for 80% power

Power analysis (post-hoc) | Power = 8.6% | -- | n = 48 for 80% power. Calculated using the formula n = [(Zα + Zβ)^2^/C^2^] + 3, where C = 0.5 × ln[(1 + |r|)/(1 − |r|)]. Comprehensive sensitivity analyses demonstrated uncertainty in the observed relationship. -- indicates not applicable or not calculated for the specific analysis method.

## Data Availability

Data Availability Statement: This study used publicly available data. Genetic data: 1000 Genomes Project (https://www.internationalgenome.org/); PRS model: PGS Catalog (PGS001308, https://www.pgscatalog.org/); Prevalence data: published literature cited in references.
